# Low Cost and Flexible UAV Deployment of Sensors

**DOI:** 10.3390/s17010154

**Published:** 2017-01-14

**Authors:** Lars Yndal Sørensen, Lars Toft Jacobsen, John Paulin Hansen

**Affiliations:** 1 Management Engineering, Technical University of Denmark, Diplomvej, Building 372, 2800 Kgs. Lyngby, Denmark; larynd@dtu.dk; 2 IT University of Copenhagen, Rued Langgaards Vej 7 2300 Copenhagen S, Denmark; latj@itu.dk

**Keywords:** UAV, drone, monitoring, multisensor, platform, software framework, beacons

## Abstract

This paper presents a platform for airborne sensor applications using low-cost, open-source components carried by an easy-to-fly unmanned aircraft vehicle (UAV). The system, available in open-source , is designed for researchers, students and makers for a broad range of exploration and data-collection needs. The main contribution is the extensible architecture for modularized airborne sensor deployment and real-time data visualisation. Our open-source Android application provides data collection, flight path definition and map tools. Total cost of the system is below 800 dollars. The flexibility of the system is illustrated by mapping the location of Bluetooth beacons (iBeacons) on a ground field and by measuring water temperature in a lake.

## 1. Introduction

The use of civilian unmanned aircraft vehicles (UAVs, or simply, drones) by professionals, researchers and hobbyists alike, has increased over the recent years. Commonly, the drones are used for aerial photography, inspection and surveys. Possible applications extend to any conceivable task where an aerial presence with minimal environmental impact brings value or new insights [1]. What sets a modern UAV platform apart from earlier flying robots is that enhancements in technology allow for the UAV to carry a networked computer payload. Consequently, the UAVs now have the ability to collect, process, store and relay data on their own and in real time.

UAVs afford three-dimensional, spatial coverage, which makes them particularly suitable for airborne sensor deployment in, for instance, ecology research and disaster management [2,3,4]. The most common deployed sensors are cameras sensitive to different light spectra. They are most useful for manual as well as automated inspection, and are often assisted by computer vision to identify relevant conditions or objects on the ground. Single drones can be programmed to follow predefined paths, covering a particular area of interest, or are flown manually either in line-of-sight, if possible, or guided by GPS or other sensory feedback. Multiple drones may be deployed in swarms, e.g., to map out and track pollution [5] or to make up a grid network and relay the data over large distances.

**Figure 1:**
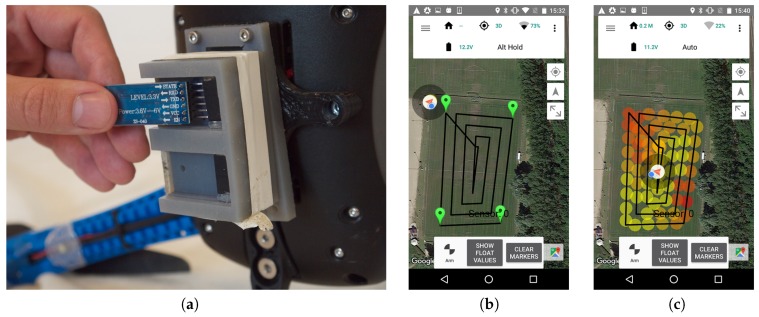
For autonomous flight: (**a**) Install sensor (Bluetooth in this scenario) and write a small sketch in Arduino, (**b**) mark the area to cover and (**c**) take-off and interpret the data (see “Supplementary files” for the data collected from this flight).

**Figure 2:**
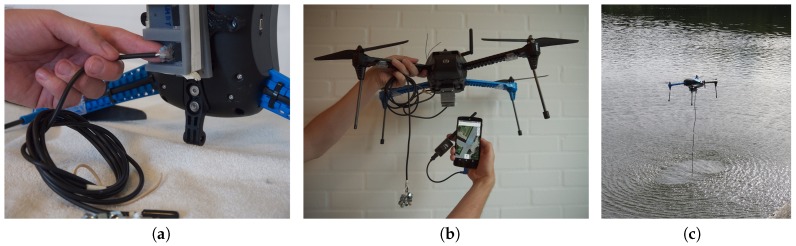
Operator-assisted flight: (**a**) A sensor (i.e., water thermometer) is mounted in the multi-socket and a small sketch in Arduino is written, (**b**) entire system with drone, sensor, smart-phone and antenna (**c**) flying manually over the lake to measure water temperature.

This paper proposes an airborne sensor platform based on commercial off-the-shelf components that provides a modularized sensor system and data acquisition infrastructure. The drone is a commercial quad-copter that allows for attaching external sensors and relaying the data back to a ground station using a telemetry communications link. The platform supports a simple and expandable interface for attaching custom sensors to the UAV, overcoming the limitation of single-purpose platforms which are costly to convert for other tasks. Since the sensor system is modularized, sensors can be exchanged rapidly. Besides the hardware platform, which easily integrates sensors, we provide the possibility to use an established open-source infrastructure to collect and visualize sensor data from the drone in real time. This allows for both autonomous path generation and following (see Figure 1) and operator-assisted (see Figure 2) flights towards areas of interest based on sensor feedback — and beyond line-of-sight if needed (The drone needs not be visible to the operator, but some line-of-sight is, however, required in the sense that telemetry signals may not be blocked, as this would result in lost data points).

Our choice of a multi-rotor aircraft delivers desirable operational parameters in some respect (i.e., agility, vertical take-off and landing, hovering and low-altitude performance), but sacrifices on other parameters like range, speed, and altitude. However, the proposed design targets makers, students and researchers that intend to conduct experiments with custom sensors and only need data collected within the boundaries of unlicensed operation. To that end, our system may be considered a prototyping platform for airborne sensor deployments, although it provides a way to rapidly implement a concrete application in its own right. In particular, we hope it may become a flexible first-step learning tool for students within engineering and environmental programmes.

While designing the system, four primary design objectives were kept in mind. The system should be: (1) low-cost; (2) easy to use; (3) modularized, for fast development and deployment of sensors; and (4) provide real-time data.

The following sections will first outline some usage scenarios. A more detailed system description follows with its technical implementation presented and illustrated with a field demonstration. Finally, we discuss shortcomings and future improvements.

## 2. Previous Work

Low-cost, light-weight UAVs have been used extensively within research for more than a decade to measure, for instance, air quality [6,7], mapping of 3D geodata [8,9,10] and remote sensing within agriculture [1,11,12,13]. These UAVs were build with a single purpose and with one particular sensor on board. While relatively cheap in comparison with other means for airborne measurements (e.g., airplanes, helicopters or high-grade UAVs), they cost several thousand dollars.

The use of open source UAV software is promoted by several research teams, (e.g., [10,14]). Aside from research, amateurs with an interest in UAVs have formed communities at places like diydrones.com to show their prototypes and discuss further developments. Some of the drones are made of parts from electronics outlets, while others are built using open-source Arduino parts intended for aviation (e.g., “Arducopters”). Anderson [15], who initiated the diydrones community, sees this as an example of amateur makers potentially revolutionizing the industry by sharing, for instance, code for a 3D-printout of a construction part. When cheap, consumer-grade drones became available, their flight computers were hacked almost immediately (e.g., [16]). Even with this "maker" movement, modifying low-cost UAVs for remote sensing is still difficult, which may prevent people with limited technical skills in computers and embedded control systems to take advantage of them.

Sensor measurements from UAVs may benefit teaching, for example, in engineering and environmental disciplines. Jung et al. [17] developed a low-cost UAV test-bed based on a model airplane, with in-house -design and -assembling of most system parts. They argue that a way to provide interdisciplinary skills for UAV development is to promote educational projects on UAV technologies. Recently, Eriksen et al. [18] and Mathias [19] augmented low-cost drones with the same educational purpose.

## 3. Usage Scenarios

The following fictitious scenarios guided our implementation. All address the collection of real-time data from the sensor attached to the drone.

- **Mapping WiFi coverage in outdoor areas**
  The task is to map out wireless network (WiFi) coverage in 2D or 3D for a large outdoor area, for instance a festival venue. A technician sets up access points and mounts a sensor on the drone that collects signal strength (i.e., received signal strength indicator (RSSI) values). A telemetry radio dongle is connected to the technicians’ smart phone on which a flight path covering the entire area is marked out using an Android ground station application. The drone will then fly between the set way-points at regular intervals. For each flight, it sends timestamped and absolute positioned sensor values back via the telemetry link. The Android app generates a heat map in real time, allowing the technician to quickly assess the coverage without waiting for the entire flight path to complete. Offline, the technician further analyses the data collected.
- **Detecting radio beacons**
  A simple Bluetooth 4.0 (BLE) module is used to detect and track radio tags, i.e., iBeacons [20]. A beacon is attached to a key-chain that has been lost on a field. The drone flies over the field and maps signal strengths, guiding the search for the key-chain.
  A signal map can be updated by flying over the area multiple times, for example, when tracking objects in motion. A zoologist tags a beacon to a badger cub. In the following weeks, the drone tracks the cub when outside its cave, mapping how its territory expands day by day.
- **Tracking pollution sources**
  An agriculturalist detects an airborne aggressive pollutant on field crops by manual inspection. The source of it needs to be tracked down quickly, and the agriculturalist, therefore, mounts a sensor for the drone platform that measures the concentration of the pollutant in the air. The drone is instructed to follow an inwards spiraling pattern from the point of detection. The agriculturalist receives values sent from the sensor as the drone continues its flight path. A heatmap pattern starts to emerge on his mobile phone, indicating a stronger concentration of pollutants in a certain direction. The agriculturalist can choose to manually alter the flight path or specify a concentration threshold for the drone sensor on the ground station software.

## 4. System Description

**Figure 3:**
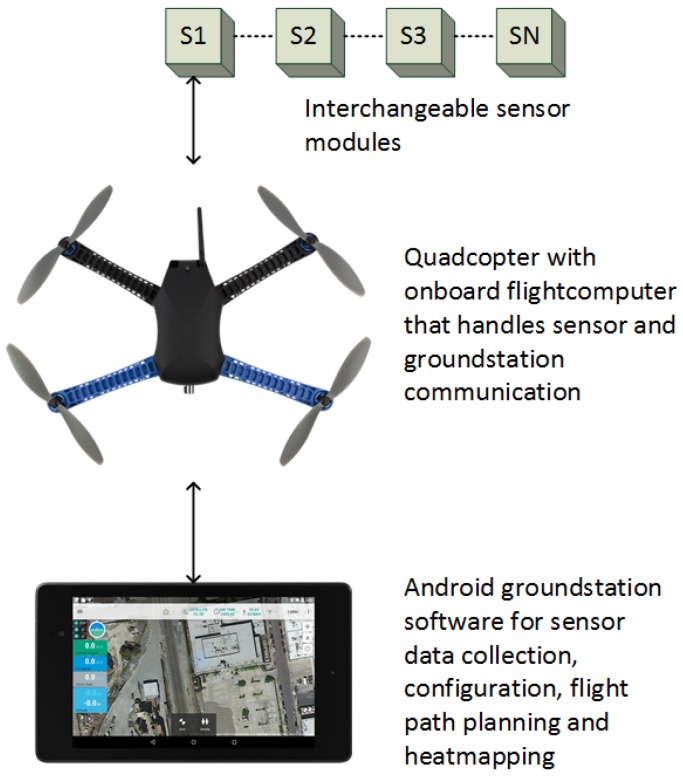
System overview. Interchangeable sensor modules attach to the quadcopter platform. Sensor data is relayed through the flight computer via a wireless telemetry link to the Android ground station application. The ground station software stores sensor data and provides intelligent survey flight path planning, heat-maps and platform configuration.

Our system is meant for attaching arbitrary low-cost sensors to an open-source robotics vehicle platform. Figure 3 provides a conceptual overview of the entire platform. The sensors are, in part, a piece of hardware that attaches to the drone. A simple protocol based on the common I2C peripheral protocol is used for interfacing with the flight computer. As long as the sensor modules conform to our hardware and software specifications, any low-weight sensor can be used.

The UAV, a 3D Robotics IRIS+ [21], which can be classified as an MAV (Micro Arial Vehicle) and a VTOL (Vertical Take-Off and Landing) according to [1], is a common commercially available drone. It was selected from a set of criteria that met our needs:

- *Cost and availability*. The drone is relatively low-cost, produced in high quantities and obtainable from resellers in both North America and Europe. Total cost for the system described herein runs just short of $800.
- *Spare-parts and repairability*. Except for the body, no legacy components are used; all spare-parts are low-cost and available from alternative brands.
- *Open-source hardware and software*. The drone is equipped with a Pixhawk flight controller [22,23]. This is a spin-off from an ETH Zürich research project [24], where the software and hardware are open-source, and the system is well-documented and supported through a community effort.
- *Telemetry options*. The drone ships with long-range radio telemetry modules. One is attached to the Pixhawk, and the other can be tethered to a computer or smart phone using a USB cable. The radio module firmware is optimized for communication using the open standard MAVLink protocol [25].
- *Attaching payloads*. The drone has mounting holes underneath the body for attaching a camera gimbal. This makes it easy to make a custom bracket for attaching other hardware without altering the body.

We prototyped a casing that attaches to a bracket that fits in the gimbal mounting points (see the first image at Figure 1). The housing contains an Arduino-based microcontroller that is powered by and interfaces with the flight computer. Smaller housings containing the actual sensors can then be attached to the microcontroller unit.

The readings picked up by the sensor module are relayed to a ground station using the featured telemetry modules; on the drone, the radio link module is connected directly to the flight computer (the Pixhawk/PX4). For this to work seamlessly with the drone setup, modifications have been made to the open-source flight computer firmware and ground station. Firstly, drivers and user space code on the Pixhawk computer interface with the sensor module and communicate with the ground station using custom messages defined as part of an existing open protocol format. Extensive modifications have gone into the code base of an open-source ground station application for Android. This allows the user to configure flight paths for surveying and retrieving the sensor data stream that, in turn, are seen in real time and are visualized on the map for every 10th reading—used later for offline analysis (see “Supplementary file” for example of collected data).

In addition, built-in safety features in the flight computer manage potentially dangerous situations; the UAV will automatically land when the battery is running low or when crossing a geofence boundary. In its current state, the drone is operational for ∼25 min on a single battery, while the Android phone (the used Android phone is a Google Nexus 5 running Android 6.0) is able to operate for ∼1 h. The Pixhawk flight computer is not solely intended for air vehicles. It may just as well be applied for autonomous path following in rovers or other ground vehicles, and our sensor platform may be ported to these vehicles without changing any parts in the system.

## 5. Technical Implementation

Our design goals call for a "full stack" implementation, touching components from sensors to the ground station. This includes an IRIS+ drone, a Pixhawk flight computer, an ArduPilot flight stack and a microcontroller driving the sensors of choice.

**Figure 4:**
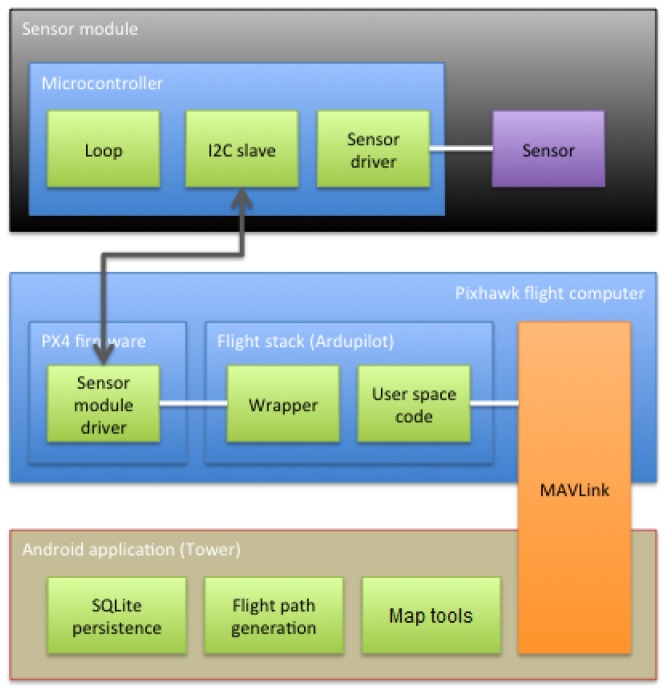
General package diagram of software involved in the system. The green packages are contributions of our package, including map tools, i.e., heat-map visualization.

Substantial previous work [22,23,26,27] has gone into creating the flight computer, its firmware and the ground station software, thus enabling us to focus on integrating the features supporting the usage scenarios. Our original contributions include building and programming sensor modules, developing drivers and communication extensions for the flight computer, and building system specific features into the Android ground station application. Figure 4 shows the overall system components and the software packages that our project has contributed.

### 5.1. Sensor Modules

**Figure 5:**
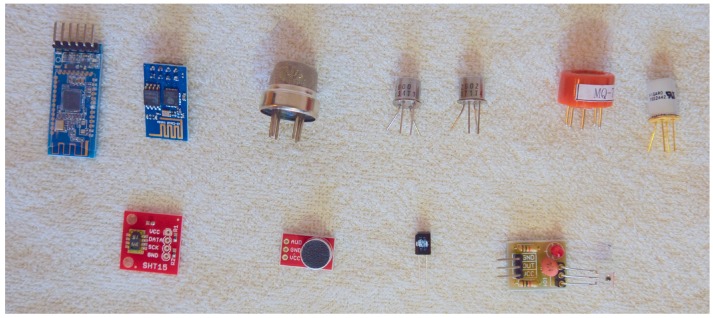
Examples of 11 low-cost sensors that can be integrated with the Arduino based micro controller. From top left are the following modules/sensors: **HM-10** (Bluetooth), **ESP8266** (WiFi), **MQ-135** (air quality: NH3, NOx, alcohol, benzene, smoke and CO${}_{2}$), **Figaro TGS2600** (air quality: methane, CO, ethanol, hydrogen and iso-butane), **Figaro TGS2602** (air quality: ammonia, hydrogen sulfide, toluene, ethanol and hydrogen), **MQ-7** (air quality: CO), **Figaro TGS2442** (air quality: CO), **SparkFun 13683** (humidity and temperature), **SparkFun 12758** (electric microphone), **IR (infrared light) receiver** and **IR receiver module**. The weight and cost of the sensors can be seen in Table 1.

To facilitate easy module installation, a bracket has been designed [28] to fit two mounting points underneath the drone, as originally intended for a camera gimbal. A sensor module can then be fitted to the bracket in various ways. It will not interfere with the structural integrity of the drone as long as the module is kept within certain physical limits: The IRIS+ specifications list a maximum payload of 400 g, and, to minimize handling interference, we suggest a sensor module design *no larger* than approximately the volume of the body of the drone (L 200 × W 120 × H 70 mm). The design of the module can be seen in image 1 of Figure 1: three parts are 3D printed to act as (i) base; (ii) container for micro controller and (iii) sensor mount. The sensor mount is for convenience to easily replace a sensor, while the base and container for the micro controller are the main parts of the system. By 3D-printing the mounts ourselves, we can comply with a broad range of sensors within a very short production time (see Figure 5 for examples), at a low cost, and just adding a few extra grams to the payload of the drone.

The Pixhawk flight controller exposes several pins useful for connecting peripherals. Besides digital GPIO (general purpose input/output) pins, there are dedicated connectors for UARTs (universal asynchronous receiver/transmitter) and SPI (serial peripheral interface)/I2C (inter-integrated circuit) buses. Since most UARTs are used for other peripherals and SPI requires a dedicated slave-select signal for each connected device, the I2C bus [29] was chosen for its availability and the possibility to daisy-chain multiple devices using just two signal wires.

The choice of I2C also means that a cable with just four wires (+5 V, Ground, Data and Clock) needs to be brought out from the flight computer to the underside of the chassis where the sensor module attaches to the mounting bracket. Power is provided by the Pixhawk as a regulated 5 V source, but the I2C clock and data lines are designed for 3.3 V logic only. The sensor module therefore must be able to accept 5 V power while honoring the 3.3 V levels on the bus lines, a common feature for several modern microcontrollers.

Although many sensor ICs support I2C directly, these are meant to be connected to an intermediary, such as a microcontroller for offloading communication and to perform preliminary data processing. This is also our strategy: a sensor module must contain at least a microntroller or embedded processor with a hardware I2C interface and support for the addressing and register scheme that we outline. The embedded computer can then connect to and retrieve readings from any number of sensor peripherals and relay the data back to the flight computer upon request in a standardized format.

At this point, we only support one sensor module at a time. This limitation is a result of not being able to handle requests for more than one value at a time from the sensor module. The module must respond when addressed using the address reserved for this purpose, and it must implement responses to the virtual registers that make up a simple protocol on top of the I2C layer (see GitHub repository [30] for details of the protocol).

**Figure 6:**
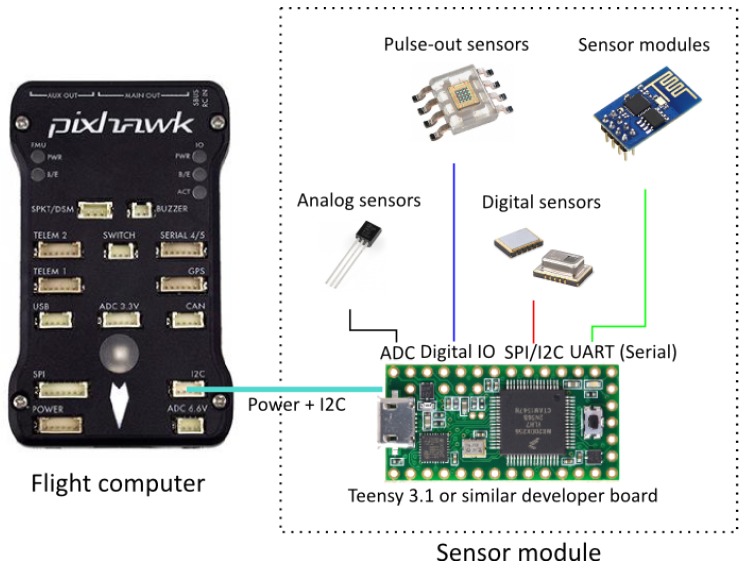
Electronic components on the drone. Basically, any type of sensor peripheral can be attached to the development board as long as it can be connected to the the Pixhawk via the I2C + power interface and implements the I2C register semantics (see code repository [30]). Photo by PixHawk/CC.

The two sensor modules we developed use a Teensy 3.1 development board [31] that contains an ARM Cortex-M4 processor running at 3.3 V. The board supports a 5V power input. Combined with a small footprint, the Teensy board is well suited for this application. The module design only occupies one I2C interface and all remaining pins (GPIO and DAC (digital to analog converter)) and bus interfaces can be used to connect the actual sensors. Figure 6 shows the electronic components involved on the drone.

If a new sensor is required, it can be implemented within hours, as the Arduino related world allows for rapidly prototyping new sensors—they just need to comply with the simple requirement that the interface is I2C at a 3.3v logic level, which many MCUs (micro controllers units) support, e.g., the entire Arduino world. If a specific MCU is needed, a voltage regulator makes it possible to use the drone’s battery as a power source, while having a logical converter to bridge between the device and the Pixhawk.

Table 1 provides cost and weight information for some of the sensors that may be attached to the drone within a few hours, while Figure 5 provides a visual of those sensors.

### 5.2. Flight Computer Firmware

In order to communicate with the sensor module, a driver for the Pixhawk firmware is needed. The software that runs on the flight computer consists of firmware on top of a real-time OS (RTOS) and a flight stack. The RTOS, based on NuttX [32], is responsible for scheduling and generally adding deterministic behavior to the timing of critical paths in the flight control software, i.e., position and attitude control. The firmware takes care of hardware abstraction, inter-module communication, and drivers. The flight stack is a set of modules or processes that perform specific tasks. These modules cooperate to get the wanted behavior from the drone. At its core, the flight stack uses inertial sensors and precise motor drivers to maintain attitude and position while responding correctly to user RC (radio controlled) inputs. These tasks have the highest priority and update frequency. Other modules with lower priority provide telemetry communication, waypoint management and more. Currently, two open-source flight stacks exist for the Pixhawk:

1. *PX4*: a highly modularized flight stack where each flight function runs in separate threads. It builds on the current NuttX and PX4 firmware development efforts.
2. *ArduPilot*: flight control runs as one large program on top of NuttX and the PX4 firwmare. The reason for this is legacy considerations. The ArduPilot flight stack can also be compiled for older integrated flight computers. In addition, it has a huge code base that supports casual and light commercial flying very well.

We have chosen the ArduPilot flight stack because it provides good support for the drone and the software ecosystem around it. From a software engineering perspective, the monolithic structure is not ideal. However, since we are running it on the Pixhawk hardware, most of the ArduPilot codes are thin wrappers around the PX4 firmware layer.

The driver that we developed for the sensor module is a native PX4 firmware driver. It is loaded by the firmware, and its update cycle is scheduled directly in NuttX. Hence, it runs autonomously from the ArduPilot flight stack, and, to access it, a wrapper driver is needed to communicate with the native driver using an *ioctl* (input/output control) like interface. This wrapper driver can then be used in the Ardupilot flight stack to read and configure the sensor module and relay data to the ground station using its own telemetry transport.

To recap, our modifications to the flight computer to support the external sensor modules are:

- *PX4 firmware native driver*: a low-level driver that accesses the I2C peripheral directly and exposes a device interface for configuration and reading data. The driver runs at a user-defined interval where it commands the sensor module to convert readings and read the actual values after an appropriate amount of time depending on the sensor.
- *Wrapper driver for the ArduPilot flight stack*: an adapter class that wraps the ioctl interface of the native driver and exposes methods that can be used directly in Ardupilot user space code.
- *ArduPilot user space code*: minor additions to the ArduPilot handles messaging to and from the sensor module drivers using a radio telemetry link.

### 5.3. MAVLink Protocol Messages

Micro Air Vehicle Link (MAVLink) is *"a very lightweight, header-only message marshalling library for micro air vehicles"* [25]. It defines an extensible set of messages and mechanisms to transfer data such as streams. The IRIS+ drone comes with MAVLink optimized radio telemetry modules, and we take advantage of this feature by describing our own MAVLink messages for sending and receiving sensor data. Because the active set of MAVLink messages must be identical on both the drone flight computer and the ground station, all messages are defined in a platform-agnostic XML file that can be used in different development projects to automatically generate the necessary message code. The code generation is done by utilities provided by the MAVlink project.

We have added two new message types to the hundreds of existing messages. The messages contain GPS position, timestamp and sensor values. Even though GPS coordinates and timestamps are sent via other messages, it is important that the sensor values are reliably correlated to the position and time of conversion. At this point, the two custom messages are just placeholders for simple single integer or floating point values. The basic structure of the sensor messages, which may be e-mailed post-flight, are: latitude, longitude, altitude in cm, sensor value, and time. e.g., 55.4868037, 12.1692884, 461.0, 27, Fri Jun 24 15:33:31 GMT+02:00 2016.

**Figure 7:**
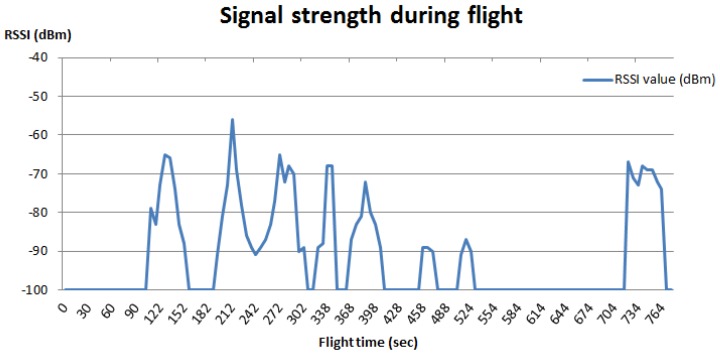
Signal strength measures during a flight above two beacons on a field. These data were collected with the drone flying at an altitude between 2 to 3 m.

To illustrate how data collection may be used for educational purposes, a student assignment could be to conduct a test of the accuracy by which the Bluetooth sensor locates a beacon on the ground (cf. Figure 2). The students would then be asked to place two beacons somewhere on a field and collect data from e.g. a 2 m altitude. First, students should make a graph of signal strengths (i.e., RSSI-values) from their test flight (cf. Figure 7). The next assignment could then be to compare these results with recordings made when flying at, for example, 10 m, to conduct low-pass filtering of the raw data [33], and, finally, to depict the relation between true distance to the beacons and the RSSI-values.

### 5.4. Android Ground Station Application

The Android application is divided into two parts because of the modularized APIs provided by 3D Robotics. The first part is the 3DR Services Library [26]. Its responsibility is to transmit and receive data between the device itself and the drone via the included radio telemetry module. This part is acting as a service on the device, providing a pipeline to other applications that need to communicate with the drone. The service will copy and store the latest of each data type coming from the drone, i.e., altitude, battery level, etc, just before notifying all subscribing applications. The subscribers may then collect the latest data in a way that prevents jeopardizing the stability of the 3DR Services Library. Our contribution to this part of the system is the ability to handle sensor values and provide these to other applications.

The second part of the Android system is the user interface (UI) named *Tower* [27], which allows the user to interact with the drone by using the 3DR Services Library. We have extended this to include the possibility to collect the sensor values and store these in a local database (the SQLite implementation for Android) from where they can be forwarded by email in a CSV (comma separated values) format. This is done pre-flight by defining the flight parameters in terms of, for example, frequency of measurements, how long each measurement is estimated to take, altitude, etc. After the flight, the email address may be defined, if not already done, and the data are then forwarded. Another feature is the possibility to show the 300 latest sensor values as a heat map, either by defining the area to be observed in the UI and letting the autopilot handle the rest, or by manually controlling the drone. In both cases, real-time data are displayed in terms of the measured value, while a graphical map is updated with every 10th data point.

If an area is marked for observation, the UI will show the flight path based on an algorithm that creates a spiral going outside-in (see Figure 1 for the process of using the drone autonomously in a scenario to locate two iBeacons in a soccer field). It is possible to easily mark an area and scan it, while still being able to override the autopilot at any given point—or not use the autopilot at all, but instead operate it manually (see Figure 2).

## 6. Discussion

The main contribution of this paper is the extensible architecture that facilitates modularized airborne sensor deployment and real-time data feedback. We expand and augment a proven embedded robotics research platform and provide a foundation for continued work on airborne sensor prototyping.

Most previous research concerning drone measurements of the environment have used computer vision (CV). Our motivation went towards a more generic sensor approach, contributing to a system that may be applied on both multi-copters, fixed-wings, rovers, and other vehicles as well. We developed a platform for of-the-shelf sensors, based on the PX4 driver, as this supports a great variety of vehicle platforms and is open-source. The PX4 system by Meier et al. [22] was intended not just for aerial vehicles but for any novel vehicle platform, and we have deliberately chosen a commercial drone based on the PX4 platform. This allows for taking our contribution to the PX4 middleware and using it in application where other types of vehicles may be more suited, be it in the air, under water or on the ground. Villa et al. [7] points to a number of limitations for the use of small lightweight UAVs in research that is critical for our system, namely short range operation, low payload capacity, and sensitivity limitations of smaller sensors. Undoubtedly, there will be research projects requiring far more than this system supports. However, the area of UAV deployment of sensors is rapidly evolving, and we believe it will take some years to sort out when to use what equipment—just like land transportation conducted with a range of vehicles, from mini-vans to long-haul trucks. Our present system offers some of the benefits that Villa et al. [7] mentions: cost effectiveness, flexibility, short time for set-up, high repeatability of data collection and safety in operation.

The used drone from 3D Robotics, which is based on the PixHawk flight computer, comes with two default telemetry systems: one for communicating with the remote control and one for communicating with other systems which can interface with the USB antenna, i.e., a computer or a smartphone. Throughout the testing and usage of the proposed system, we experienced that the USB signal was rather weak, compared to the remote control signal. At a distance of roughly 100 m, the signal strength was occasionally too weak.

In its current state, the proposed system does not support the recovery of a missed data package. Thus, a measurement may be permanently lost if the drone moves out of the telemetric field of the ground station, which also is prohibited in several countries.

A key aspect for the project was uncovering the benefits and possibilities of the system. We devised three usage scenarios that the platform should ultimately support. A weakness of this approach is the lack of proper validation of the scenarios. Are they credible to potential users and how should they be evaluated under real task conditions?

The community around research and DIY drones is thriving, and open designs and software for UAVs are becoming increasingly available. This makes it harder to maintain a plug-and-play solution for a particular brand of drone. One could picture at least two paths for future efforts: one solution is to simply ignore the drone and flight systems and make a go for an isolated and autonomous payload. Another path is to support a complete open design for a research drone system that is tailored for carrying generic sensor modules. It is our hope and ambition that we have contributed to the latter approach by the work presented in this paper and by the available source code.

## 7. Future Work

To prevent data from getting lost, the firmware on the drone should be improved to expect a confirmation message from the ground station upon retrieval of a measurement. In case the confirmation signal is absent, the measurement should be stored until the connection is re-established.

At the moment, the system is unable to accurately track a moving source in real time. However, since the system can collect and store all measured values, with some further work, it might eventually track down a source in motion. This requires an algorithm for determining the vehicles’ next most optimal heading, while the system already supports a change of way-points in mid-air.

The current usage scenarios are outdoor. However, we foresee a range of indoor sensing tasks in which drones—flying or driving—may do well, for instance locating gas leaks or mapping signal strengths in a building. Our future research will address indoor navigation by use of various non-vision sensors, e.g., ultra-sound, IR-beacons and by fingerprinting ubiquitous radio waves. In order to do this, we need a platform that can change sensors easily and quickly. Eventually, the current platform should be improved in order to carry more than one sensor at a time.

## 8. Conclusions

We have demonstrated the feasibility of turning a commercial drone into an extensible airborne sensor platform by adding new functionality to the stack of components in a drone system, from the sensor attachment to the user interface in the ground station software. The one important premise for this to succeed, however, is the availability of well-documented open-source or open-API software (and hardware for that matter) in all of the subsystems.

## Figures and Tables

**Table 1:** Examples of sensors, including cost and weight, which may be used with the proposed system. The sensors can be seen in Figure 5.

Sensor	Price	Weight	Notes
HM-10	2.92 USD	4 g	**Bluetooth** Low Energy (BLE) 4.0 module for iBeacon detection
ESP8266	1.90 USD	2 g	**WiFi** module, that i.e., can measure signal strength
MQ-135	1.70 USD	4 g	For **air quality**: NH3, NOx, alcohol, benzene, smoke and CO${}_{2}$
Figaro TGS2442	18.19 USD	2 g	For **air quality**: CO
Figaro TGS2600	15.71 USD	2 g	For **air quality**: Mehtane, CO, ethanol, hydrogen and iso-butane
Figaro TGS2602	15.52 USD	2 g	For **air quality**: Ammonia, hydrogen sulfide, toluene, ethanol and hydrogen
MQ-7	7.25 USD	2 g	For **air quality**: CO
SparkFun 13683	41.95 USD	2 g	**Humidity and temperature**
SparkFun 12758	5.95 USD	2 g	Electric **microphone** (noise filtering *will* be needed)
IR receiver	2.04 USD	2 g	Just the **IR** component. (Price includes emitter)
IR receiver module	1.29 USD	2 g	**IR** light module

## Supplementary Materials

Supplementary File 1

## References

[1] Watts A.C., Ambrosia V.G., Hinkley E.A. (2012). Unmanned Aircraft Systems in Remote Sensing and Scientific Research: Classification and Considerations of Use. Remote Sens..

[2] Erman A.T., Hoesel L., Havinga P., Wu J. (2008). Enabling mobility in heterogeneous wireless sensor networks cooperating with UAVs for mission-critical management. IEEE Wirel. Commun..

[3] Kerle N., Heuel S., Pfeifer N. (2008). Real-Time Data Collection and Information Generation Using Airborne Sensors.

[4] Quaritsch M., Kruggl K., Wischounig-Strucl D., Bhattacharya S., Shah M., Rinner B. (2010). Networked UAVs as aerial sensor network for disaster management applications. e & i Elektrotech. Inform..

[5] White B.A., Tsourdos A., Ashokaraj I., Subchan S., Zbikowski R. (2008). Contaminant cloud boundary monitoring using network of UAV sensors. IEEE Sens. J..

[6] Alvarado M., Gonzalez F., Fletcher A., Doshi A. (2015). Towards the development of a low cost airborne sensing system to monitor dust particles after blasting at open-pit mine sites. Sensors.

[7] Villa T.F., Gonzalez F., Miljievic B., Ristovski Z.D., Morawska L. (2016). An Overview of Small Unmanned Aerial Vehicles for Air Quality Measurements: Present Applications and Future Prospectives. Sensors.

[8] Bendea H., Chiabrando F., Tonolo F.G., Marenchino D. Mapping of archaeological areas using a low-cost UAV. The Augusta Bagiennorum test site. In Proceedings of the XXI International CIPA Symposium.

[9] Neitzel F., Klonowski J. Mobile 3D mapping with a low-cost UAV system. Proceedings of the International Archives of the Photogrammetry, Remote Sensing and Spatial Information Sciences, Vol. XXXVIII-1/C22 UAV-g 2011, Conference on Unmanned Aerial Vehicle in Geomatics.

[10] Niethammer U., Rothmund S., Schwaderer U., Zeman J., Joswig M. Open source image-processing tools for low-cost UAV-based landslide investigations. Proceedings of the International Archives of the Photogrammetry, Remote Sensing and Spatial Information Sciences, Vol. XXXVIII-1/C22 UAV-g 2011, Conference on Unmanned Aerial Vehicle in Geomatics.

[11] Gonzalez F., Castro M.P., Narayan P., Walker R., Zeller L. (2011). Development of an autonomous unmanned aerial system to collect time-stamped samples from the atmosphere and localize potential pathogen sources. J. Field Robot..

[12] Xiang H., Tian L. (2011). Development of a low-cost agricultural remote sensing system based on an autonomous unmanned aerial vehicle (UAV). Biosyst. Eng..

[13] Bareth G., Aasen H., Bendig J., Gnyp M.L., Bolten A., Jung A., Michels R., Soukkamäki J. (2015). Low-weight and UAV-based hyperspectral full-frame cameras for monitoring crops: Spectral comparison with portable spectroradiometer measurements. Photogramm.-Fernerkund.-Geoinform..

[14] Jang J.S., Liccardo D. Automation of small UAVs using a low cost MEMS sensor and embedded computing platform. Proceedings of the 2006 IEEE/AIAA 25TH Digital Avionics Systems Conference.

[15] Anderson C. (2013). Makers: The New Industrial Revolution.

[16] Childers B. (2014). Hacking the Parrot A.R. Drone. http://dl.acm.org/citation.cfm?id=2631585.2631586.

[17] Jung D., Levy E., Zhou D., Fink R., Moshe J., Earl A., Tsiotras P. Design and development of a low-cost test-bed for undergraduate education in UAVs. Proceedings of the 44th IEEE Conference on Decision and Control.

[18] Eriksen C., Ming K., Dodds Z. (2014). Accessible Aerial Robotics. J. Comput. Sci. Coll..

[19] Mathias H.D. (2016). An Autonomous Drone Platform for Student Research Projects. J. Comput. Sci. Coll..

[20] Cavallini A. iBeacon Bible 2.0. https://meetingofideas.files.wordpress.com/2014/06/ibeacon-bible-2-0.pdf.

[21] IRIS + Drone Specifications. https://store.3drobotics.com/products/iris.

[22] Meier L., Honegger D., Pollefeys M. PX4: A Node-Based Multithreaded Open Source Robotics Framework for Deeply Embedded Platforms. Proceedings of the 2015 IEEE International Conference on Robotics and Automation (ICRA).

[23] Meier L., Tanskanen P., Fraundorfer F., Pollefeys M. PIXHAWK: A system for autonomous flight using onboard computer vision. Proceedings of the 2011 IEEE International Conference on Robotics and Automation (ICRA).

[24] Pixhawk project at ETH Zürich. https://pixhawk.ethz.ch/.

[25] MAVLink Project Website. http://qgroundcontrol.org/mavlink/start.

[26] 3DR-Services-Library. https://github.com/ne0fhyk/3DR-Services-Library.

[27] DroidPlaner-Tower. https://github.com/DroidPlanner/Tower.

[28] Bracket for mounting the sensor module on the drone. http://www.thingiverse.com/thing:435675.

[29] Semiconductors N. (2007). I2C-bus specification and user manual. Rev.

[30] Complete Source Code for the Project Found on Github. https://github.com/Yndal/ArduPilot-SensorPlatform.

[31] Teensy Development Board. https://www.pjrc.com/teensy/index.html.

[32] NuttX Real-Time Operating System. http://nuttx.org/.

[33] Jung J., Kang D., Bae C. Personal Computing Platform Research Team Distance Estimation of Smart Device Using Bluetooth. Proceedings of the ICSNC 2013: The Eighth International Conference on Systems and Networks Communications.

